# Chondroitin sulfate supplementation improves clinical outcomes in a murine model of necrotizing enterocolitis

**DOI:** 10.14814/phy2.15819

**Published:** 2023-09-11

**Authors:** Krishna Manohar, Brian D. Hosfield, Fikir M. Mesfin, Cameron Colgate, William Christopher Shelley, Jianyun Liu, Lifan Zeng, John P. Brokaw, Troy A. Markel

**Affiliations:** ^1^ Department of Surgery Indiana University School of Medicine (IUSM) Indianapolis Indiana USA; ^2^ Riley Hospital for Children at Indiana University Health Indianapolis Indiana USA; ^3^ Department of Biochemistry and Molecular Biology IUSM Indianapolis Indiana USA

**Keywords:** chondroitin sulfate, eNOS, intestinal dysbiosis, necrotizing enterocolitis

## Abstract

Necrotizing enterocolitis (NEC) continues to be a devastating disease in preterm neonates and has a paucity of medical management options. Chondroitin sulfate (CS) is a naturally occurring glycosaminoglycan (GAG) in human breast milk (HM) and has been shown to reduce inflammation. We hypothesized that supplementation with CS in an experimental NEC model would alter microbial diversity, favorably alter the cytokine profile, and (like other sulfur compounds) improve outcomes in experimental NEC via the eNOS pathway. NEC was induced in 5‐day‐old pups. Six groups were studied (*n* = 9–15/group): (1) WT breastfed and (2) Formula fed controls, (3) WT NEC, (4) WT NEC + CS, (5) eNOS KO (knockout) NEC, and (6) eNOS KO NEC + CS. Pups were monitored for clinical sickness score and weights. On postnatal day 9, the pups were killed. Stool was collected from rectum and microbiome analysis was done with 16 s rRNA sequencing. Intestinal segments were examined histologically using a well‐established injury scoring system and segments were homogenized and analyzed for cytokine profile. Data were analyzed using GraphPad Prism with *p* < 0.05 considered significant. CS supplementation in formula improved experimental NEC outcomes when compared to NEC alone. CS supplementation resulted in similar improvement in NEC in both the WT and eNOS KO mice. CS supplementation did not result in microbial changes when compared to NEC alone. Our data suggest that although CS supplementation improved outcomes in NEC, this protection is not conferred via the eNOS pathway or alteration of microbial diversity. CS therapy in NEC does improve the intestinal cytokine profile and further experiments will explore the mechanistic role of CS in altering immune pathways in this disease.

## 1 INTRODUCTION

Necrotizing enterocolitis (NEC) remains a devastating and morbid condition in premature and low birth weight neonates and is associated with mortality rates as high as 20%–30% (Jacob et al., 2015). The pathophysiology of this disease is multifactorial and is thought to be driven by an immature intestine and immune system, microbial dysbiosis, and formula feeding—all of which subsequently drive pathogenic inflammatory responses (Bazacliu & Neu, 2019; Neu & Walker, 2011) that can result in intestinal necrosis and the need for surgical intervention. Targeted medical therapy is limited, particularly when the disease progresses to requiring surgery (Knowles et al., 2021; Neu & Walker, 2011). One well‐established factor to prevent the development of NEC is human breast milk (HM) (Altobelli et al., 2020; Meinzen‐Derr et al., 2009), as exclusively formula‐fed infants have up to 6–10 times increased incidence of NEC (Lucas & Cole, 1990; Miller et al., 2018). The protective effects of HM are hypothesized to be secondary to alterations of the gut microbiome as well as the delivery of many compounds that have immune‐modulating activities (Institute of Medicine Committee on the evaluation of the addition of ingredients new to infant F, 2004; Knowles et al., 2021; Underwood, 2013). However, recent data suggest that disparities such as ethnicity, race, and socioeconomic status can result in decreased access to both mother's breast milk and banked donor milk (Boundy et al., 2017). Therefore, it is prudent to understand the components of HM that help reduce the risk of NEC so that formula supplementation can occur if needed.

HM contains many protective factors including secretory IgA, lactoferrin, and various oligosaccharides including glycosaminoglycans (GAGs; Altobelli et al., 2020; Knowles et al., 2021). These molecules, usually sulfated and consisting of repeating disaccharide units, are highly abundant and can serve as prebiotics for commensal bacteria in the intestine (Coppa et al., 2011). They have been shown to exhibit immune‐modulatory effects in various disease processes, participate in cell‐surface receptor signaling (Bering, 2018; Burge et al., 2020; Coppa et al., 2011), and may have the ability to act as a soluble receptor to prevent the binding of pathogens and pathogen‐associated proteins (Burge et al., 2019; Coppa et al., 2012; Knowles et al., 2021). A prominent GAG gaining clinical interest in the treatment of NEC is chondroitin sulfate (CS), which comprises over half of the normal GAG content in human milk (Burge et al., 2020; Knowles et al., 2021) and is nonexistent in most major infant formulas (Institute of Medicine Committee on the evaluation of the addition of ingredients new to infant F, 2004). Additionally, the concentration of CS in human breast milk is higher in preterm mothers than in term mothers, thereby suggesting some evolutionary importance for this compound to preterm infants (Burge et al., 2019; Coppa et al., 2012; Knowles et al., 2021).

The exact therapeutic effect of CS is unknown; however, CS has been shown to have anti‐inflammatory, immune‐modulatory, and prebiotic properties (Knowles et al., 2021; Martel‐Pelletier et al., 2015; Shmagel et al., 2019). Additionally, GAGs, and specifically CS, have been implicated in being a key component of the extracellular matrix which is lost in the intestines of neonates during NEC (Ade‐Ajayi et al., 1996). GAGs have sulfated residues that are believed to participate and regulate various biological functions (Anower & Kimata, 2015). CS can be a complex heterogenous polysaccharide with various sulfating patterns (du Souich et al., 2009; Martel‐Pelletier et al., 2015), but usually when extracted and processed from the cartilage of bovine, porcine, and galline sources, has single sulfate moieties (Martel‐Pelletier et al., 2015; Volpi, 2007). In human milk studies, Coppa et al. found that in mothers of healthy‐term infants, CS comprised the largest proportion of GAG's (56%) as compared to bovine milk which only contained 21% CS. However, the samples contained equal proportions of sulfated CS (Coppa et al., 2011; Martel‐Pelletier et al., 2015).

The exact mechanisms of CS's effect on the microbiome are unknown, however, CS appears to decrease the invasion of pathogenic bacteria across the gut wall and promote the growth of commensal bacteria (Burge et al., 2019). A systematic review of studies looking at the effects of CS on the gut microbiome suggested that CS supplementation did not have clear effects on microbial diversity, but overall did improve the abundance of specific beneficial genera of bacteria such as *Bacteroides and Bifidobacterium* (Shmagel et al., 2019), and specifically increased some populations of sulfate‐reducing bacterial populations such as *Desulfovibrio, Desulfobacteria*, and *Desufobacter* (Dordević et al., 2021; Shmagel et al., 2019). It has been demonstrated that chondroitin sulfate has increased H_2_S in mice endogenously by increasing the relative abundance of sulfate reducing bacteria in the gut (Chen et al., 2022; Pichette et al., 2017), further providing support for the importance of sulfur‐residue signaling. Additionally, previous studies from our laboratory have shown that multiple sulfur donor compounds exerted their beneficial properties through the endothelial nitric oxide synthase (eNOS) pathway by improving mesenteric perfusion and decreasing intestinal injury (Drucker, Jensen, Ferkowicz, & Markel, 2018; Hosfield et al., 2022). Endothelial cells in the intestine express endothelial nitric oxide synthase (eNOS). Sulfur donors, including NaHS and GYY4137 are theorized to interact with this enzyme to promote eNOS dimerization and increase nitric oxide production which acts to improve mesenteric perfusion and prevent intestinal injury (Drucker, Jensen, Ferkowicz, & Markel, 2018; Hosfield et al., 2022; Jensen et al., 2017).

We therefore hypothesized that formula supplementation of chondroitin sulfate would improve outcomes and decrease intestinal inflammatory cytokines in experimental NEC. Additionally, we hypothesized that these benefits would be mediated through the eNOS pathway and by the improvement of microbial dysbiosis and diversity.

## 2 METHODS

### 2.1 Animals

The Indiana University Institutional Animal Care and Use Committee (IACUC) approved all animal use (Protocol #19122). C57BL/6J (WT) and B6.129P2‐Nos3^tm1Unc^/J eNOS KO (eNOS knockout) mice were purchased from Jackson Laboratories and mouse pups were bred in‐house. Due to the young age and sexual immaturity of the pups at the time of the study (5–9 days old), both male and female pups were used collectively, and sex was not considered a factor during statistical analysis. The eNOS KO mice were validated in our laboratory by PCR in a prior study (Hosfield et al., 2022).

### 2.2 Necrotizing enterocolitis model

Necrotizing enterocolitis was induced in mice as previously described (Drucker, Jensen, Ferkowicz, & Markel, 2018; Drucker, Jensen, Te Winkel, et al., 2018; Hosfield et al., 2022; Zani et al., 2008). In brief, experimental pups were separated from their mothers on postnatal day 5 (p5) and placed in satellite housing in a temperature and humidity‐controlled incubator (32°C and 40% humidity). Breastfed control pups remained with their mothers to be breastfed. Multiple litters comprised each experimental group. Six groups were studied (*n* = 9–15/group): (1) WT breastfed controls (BF Ctrl), (2) Formula‐fed controls (FF Ctrl), (3) WT NEC (NEC), (4) WT NEC + 200 mg/kg CS in formula (NEC + CS), (5) eNOS knockout NEC (eNOS KO NEC), and (6) eNOS knockout NEC + 200 mg/kg CS in formula (eNOS KO NEC + CS). In each of these groups, daily weights were obtained. A batch of formula was freshly prepared every 48 h using 4 g Esbilac milk replacer (PetAg, Hampshire, IL |SKU# 26689), 6 g of Similac Advance powder (Similac, Columbus, OH| Model # 746696EA) that was mixed together in 20 mL of deionized water and stored at 4°C. Each day, formula was made based on daily weights and the caloric needs of the pups from the batch stock and supplemented with 0.008 mg/g of Lipopolysaccharide (LPS) isolated from *Escherichia Coli* O111:B4 (cat # L4391, Sigma‐Aldrich Company, St. Louis, MO). Those in the group to receive chondroitin sulfate, had their formula supplemented to receive 0.2 mg/g chondroitin sulfate daily (Cimini et al., 2022; Gao et al., 2022; Qi et al., 2021; Singh et al., 2021; Song et al., 2017). Experimental pups were gavage fed a hyper‐concentrated formula (300 kcal/kg/day) three times per day using a 1.9 French catheter. As a reference, the average NICU premature infant receives 150 kcal/kg/day (Chen et al., 2019). Prior to each feed, pups were placed in a hypoxia chamber with 5% oxygen and balanced nitrogen for 10 min. Additionally, they were exposed to hypothermia at 4°C for 12 min after the first and last feeds of the day. Pups were monitored daily for clinical sickness scores and weight gain. On postnatal day 9 (p9), the pups were humanely killed via cervical decapitation, intestinal tissue was harvested, and stool was collected from the rectum for further analysis. Mice who succumbed prior to reaching p9 were excluded from analysis except for in mortality curves.

### 2.3 Chondroitin sulfate concentration in milk and formula

An IRB was obtained through Indiana University (IRB protocol # 20114) and consent was provided from volunteers. No private data or medical records were accessed. Milk samples were de‐identified and stored. All procedures followed were in accordance with the principles of the Declaration of Helsinki.

Human breast milk was collected from three volunteers of term infants. Three additional samples of banked donor breast milk were also collected from Riley Children's Hospital's milk laboratory that were collected from mothers of term infants. Four standard formula samples were also assessed including Nutramigen hypoallergenic formula, Similac 360 Total Care, Similac Pro‐Total Comfort, and Similac Special Care Premature. Milk samples were centrifuged down and the top fatty layer was removed. All samples were analyzed for chondroitin sulfate content using an All‐species Chondroitin Sulfate sandwich ELISA kit (LS Bio| LS‐#F55475). Samples were assessed with a Spectramax M2 Microplate Reader (Molecular Devices) at 560 nm. The ELISA was performed in triplicate and pooled and analyzed.

### 2.4 Exogenous chondroitin sulfate

Chondroitin sulfate was obtained from Bulk Supplements (SKU# CHON100). This was sourced from bovine cartilage. An analysis of chondroitin sulfate purity was performed on Agilent 1290 LC‐6545 via the quadrupole time of flight (QTOF) mass spectrometer to assess for the identity of chondroitin sulfate under negative ionization mode. The non‐sulfated CS under negative mode was m/z 378, the single‐sulfated CS compound was at m/z 458, and the double sulfated CS was at m/z 268. A C18 HPLC column was used for separation. 0.1% ammonia bicarbonate in water and acetonitrile were used as the mobile phase. The purity of chondroitin was established by a UV detector under 194 nm. A certificate of analysis was provided by the company for the lot number used in these experiments. The dose of chondroitin sulfate was determined by a literature search of chondroitin sulfate doses in different rodent models of disease that all used a dose of 200 mg/kg for beneficial therapeutic effects (Cimini et al., 2022; Gao et al., 2022; Qi et al., 2021; Singh et al., 2021; Song et al., 2017).

### 2.5 Clinical assessment

Clinical status was assessed on all experimental pups three times daily. Pups were weighed daily and percent weight gain was analyzed and compared from p5 to p9 to account for differences in initial pup weight. Breastfed controls were scored on p5 and p9. The scoring system has been used and validated previously in our laboratory (Drucker, Jensen, Ferkowicz, & Markel, 2018) and measures on a scale of 0–3 the pup's hydration status, activity, alertness, and body color. These parameters are added up for a final score ranging from 0 to 12, with a score of zero representing a healthy, hydrated, and alert pup. The reported clinical sickness scores in our analysis were the pup's score before killing by cervical decapitation on p9 minus the baseline score on p5.

### 2.6 Macroscopic intestinal injury score

After killing, the intestine was harvested and inspected and scored from 0 to 2 on consistency, color, and dilatation as described previously (Drucker, Jensen, Ferkowicz, & Markel, 2018). Scores were reported as the sum of each component, for a maximum intestinal injury score of 6. Briefly, a score of 0 in each category represented an intestinal segment that was normal in consistency with no discoloration or dilation. A score of 2 in each category (and a sum of 6), represented the intestine that was extremely friable and “jelly‐like,” with extensive discoloration/dilation, and signs of necrosis.

### 2.7 Microscopic intestinal injury score

After euthanasia, terminal ileal samples were fixed in 4% paraformaldehyde for 24 h and then dehydrated in 70% ethanol. The tissues were embedded in paraffin, sectioned, and stained with hematoxylin and eosin. Images of these segments were taken with a Leica DM300 (SKU# 13613384) microscope at 10× magnification, and four to five images were captured of each intestinal segment from each animal. The histological injury was scored on a scale from 0 to 4 as previously described (Drucker, Jensen, Ferkowicz, & Markel, 2018) where a score of “0” represents normal intestine with intact architecture; “1” represents some alteration of enterocytes and the presence of more clear cells; “2” represents significant derangement of villus enterocytes and structure, “3” represents loss of villi and epithelial sloughing; and “4” represents bowel necrosis +/− perforation. Scoring was blindly performed by two independent researchers and reported as an average of the two scores.

### 2.8 Stool analysis

At killing, stool was expressed from descending colon/rectum, snap frozen in liquid nitrogen, and stored at −80°C. Intestinal microbiome analysis was performed via 16 s rRNA gene sequencing as described previously (Hosfield et al., 2020). In brief, after DNA extraction, amplification was performed by using the Shoreline Complete V4 Kit (Cat # SCV4, Shoreline Biome). DNA was then sequenced on an iSeq 100 with 150‐bp paired‐end reads and were curated using Mothur Software (University of Michigan). Data were screened for sequencing errors and aligned to the SILVA bacterial SSU reference database (Max Planck Institute for Marine Microbiology and Jacobs University, Bremen, Germany). Finally, sequences were then screened for chimeras and classified using the Greengenes database (Second Genome Inc.). The top 10 bacterial families were analyzed from the stool samples and expressed as a percentage out of 100% of the total bacterial families in the stool sample. Alpha diversity was measured using the Shannon index. NMDA (nonmetric multidimensional) scaling was employed as a method to compare the Bray–Curtis dissimilarity values.

### 2.9 Cytokine analysis

Terminal ileal segments were explanted and placed in conical tubes with RIPA (radioimmunoprecipitation assay) buffer (Millipore Sigma). Tissue was then homogenized using an ultrasonicator. Tubes were centrifuged and cell lysates were removed and stored separately at −80°C. When ready, cell lysates were used to measure cytokine levels via multiplex beaded assay (EMD Millipore | #MTH17MAG‐47). To estimate total protein concentration in samples, we used a BCA Protein Assay Kit (ThermoFisher Scientific Catalog # J63283.QA). These experiments were repeated in duplicate.

### 2.10 Mortality curves

Mortality curves were created from animals from multiple different similar experiments undergoing the same protocol as above were combined in the four conditions of interest: (1) BF Controls (*n* = 32), (2) FF controls (*n* = 15), (3) NEC (*n* = 33), and (4) NEC + CS (*n* = 21).

### 2.11 Statistical analysis

GraphPad Prism 9 (GraphPad Software) was used for statistical analysis and creation of figures. Data were tested for normality using the Shapiro–Wilk and Kolmogorov–Smirnov tests. Continuous and normally distributed data were expressed as mean ± standard deviation (SD). Ordinal data and non‐normally distributed data were expressed as median and interquartile range (IQR). Data were analyzed with t‐test, Mann–Whitney *U*‐test, Kruskal–Wallis, and ANOVA where appropriate. For Kruskal–Wallis and ANOVA comparisons, if significance was found, multiple comparisons were performed with Dunn's or Sidák's ad hoc correction. Mortality curves were compared by comparing two curves at a time using the Gehan–Breslow–Wilcoxon test. Interpretation of significant p‐value for multiple comparisons was made manually using the Bonferroni method with four comparisons of interest for an adjusted *p* <0.0083. A *p* <0.05 was considered statistically significant for all other analysis. A PERMANOVA test was used to analyze the NMDS plot and differences between the Bray–Curtis dissimilarity values between groups.

## 3 RESULTS

### 3.1 Experimental NEC yields worse outcomes than breast and formula‐fed controls

**FIGURE 1:**
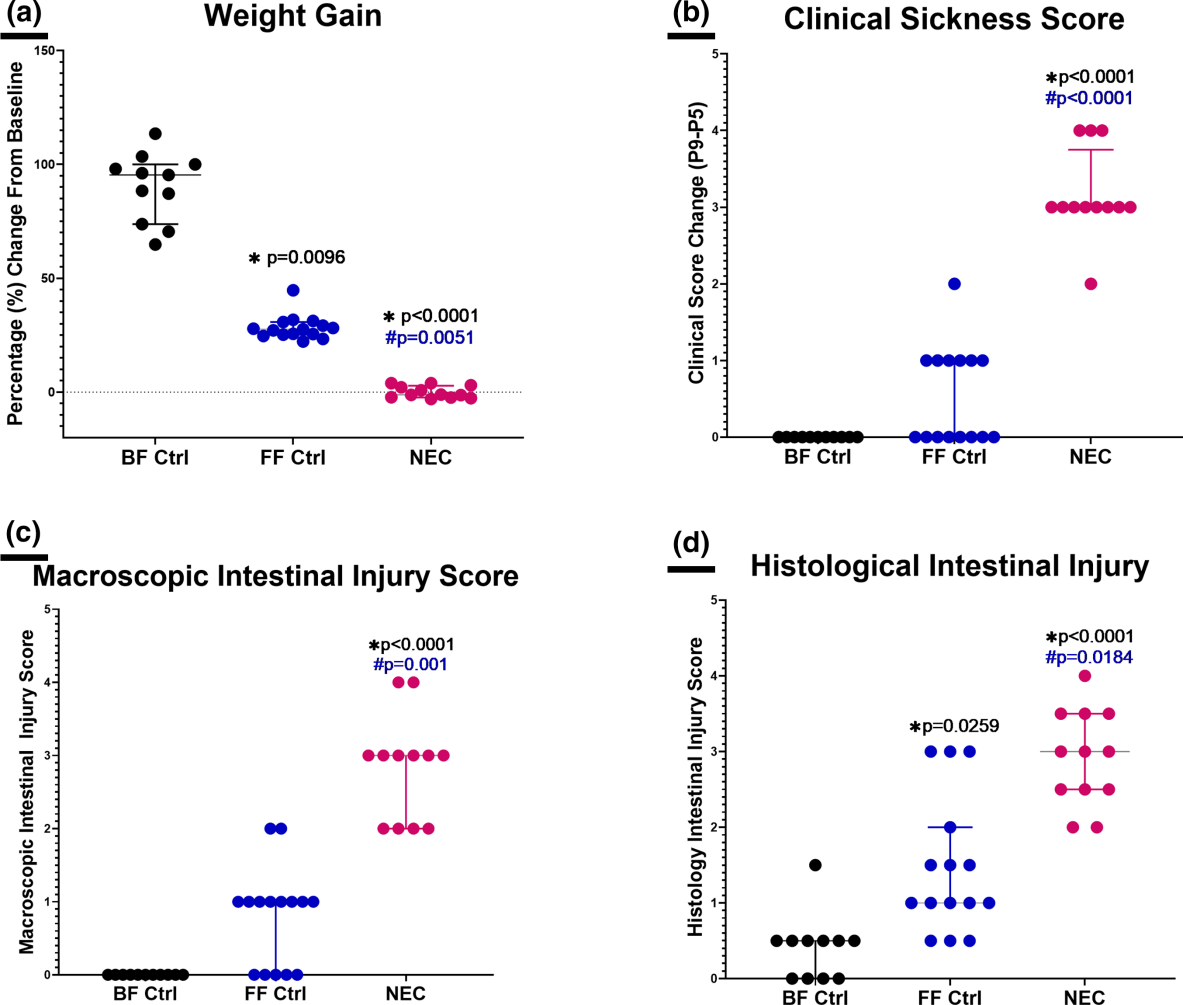
Breastfed controls (BF Ctrl, *n* = 11) versus formula‐fed controls (Ff Ctrl, *n* = 15) versus necrotizing enterocolitis (NEC, *n* = 12). (a) Weight Change: Mice with NEC showed a significant lack of weight gain compared to BF Ctrl (*p < 0.0001) and FF Ctrl (#*p* = 0.0051). FF Ctrl showed a significant difference (*p = 0.0096) compared to BF Ctrl. (b) Clinical Sickness Score Change: Mice with NEC showed a significant worsening of clinical sickness scores when compared to BF Ctrl (*p < 0.0001) and FF Ctrl (#*p* = 0.0001) with no significance between BF Ctrl and FF Ctrl. (c) Macroscopic Gut Injury Score: Mice with NEC showed a significant worsening of macroscopic gut injury scores when compared to BF Ctrl (*p < 0.0001) and FF Ctrl (#*p* = 0.0010) with no significance between BF Ctrl and FF Ctrl. (d) Microscopic Intestinal Histology Score: Mice with NEC showed a significant worsening in microscopic intestinal histology scores compared to BF Ctrl (*p < 0.0001) and FF Ctrl (#*p* = 0.0184). FF Ctrl showed a significant worsening (*p = 0.0259) compared to BF Ctrl. All analyses performed were Kruskal–Wallis with Dunn's multiple comparisons test.

When compared to breastfed controls (*n* = 11), mice undergoing the NEC experimental model (*n* = 12) showed a significant lack of weight gain (BF Ctrl = 95.38 [73.77–100], NEC = −1.163 [−2.358–2.802], *p < 0.0001, Figure 1a), worse clinical sickness scores (BF Ctrl = 0[0–0], NEC = 3 [3–3.75], *p < 0.0001, Figure 1b), higher macroscopic intestinal injury scores (BF Ctrl = 260 0[0–0], NEC = 3 [2–3], *p ≤ 0.0001, Figure 1c *)*, and higher histological intestinal injury scores (BF Ctrl = 0.5[0–0.5], NEC = 3 [2.5–3.5], *p* < 0.0001, Figure 1d).

When compared to formula‐fed controls (*n* = 15), mice with NEC also showed a significant lack of weight gain (FF Ctrl = 27.62 [25.21–30.77], NEC = −1.163 [−2.358–2.802], #*p* = 0.0051, Figure 1a), worsened clinical sickness scores (FF Ctrl = 0[0–1], NEC = 3 [3–3.75], #*p* = 0.0001, Figure 1b), higher macroscopic intestinal injury scores (FF Ctrl = 1[0–1], NEC = 3 (Bazacliu & Neu, 2019; Neu & Walker, 2011), #*p* = 0.001, Figure 1c), and higher histological intestinal injury scores (FF Ctrl = 1 (Bazacliu & Neu, 2019; Jacob et al., 2015), NEC = 3 [2.5–3.5], #*p* = 0.0184, Figure 1d).

When compared to breastfed controls, formula‐fed controls showed a significantly lower weight gain (*p = 0.0096, Figure 1a) and higher histological intestinal injury scores (*p = 0.0259, Figure 1d), but no significant differences in clinical scores or macroscopic intestinal injury scores.

### 3.2 Chondroitin sulfate content in human milk and formulas

**FIGURE 2:**
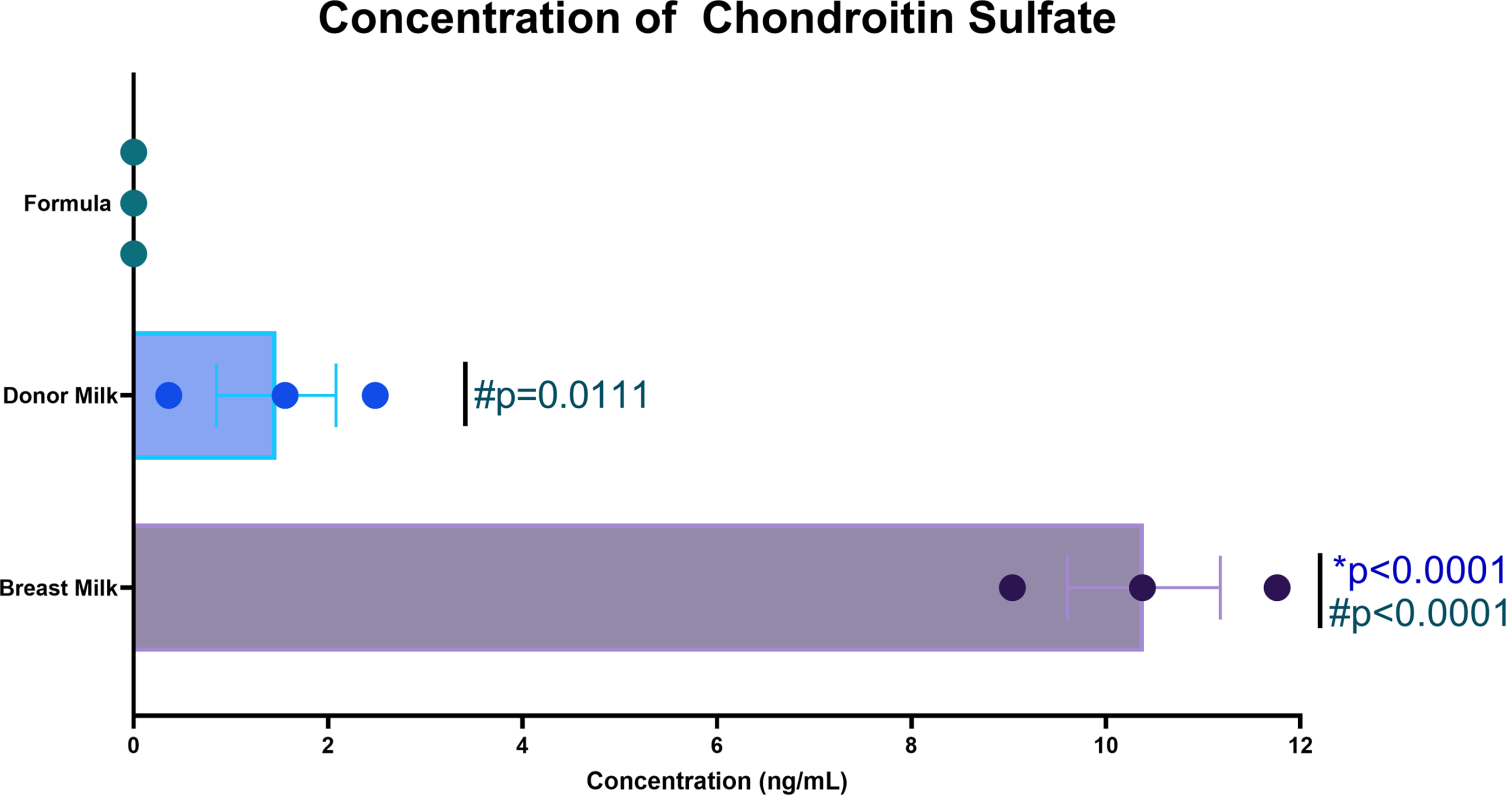
Content of chondroitin sulfate in milk sources. CS in breast milk (*n* = 6), donor milk (*n* = 6), and formula (*n* = 7) were pooled and compared. Breast milk CS content 7.941 ± 1.18 is significantly higher were compared to both donor milk (*p* < 0.0001; 1 ± 0.41) and formula (*p* < 0.0001; 0 ± 0). Donor milk CS content was also significantly higher than formula (*p* = 0.0111). Two‐way ANOVA was performed with Tukey's multiple comparisons test.

The content of CS in breast milk, donor milk, and formula were compared (Figure 2). Breast milk CS content (7.941 ± 1.18 ng/mL) was significantly higher when compared to both donor milk (*p < 0.0001; 1 ± 0.41 ng/mL) and formula (#*p* < 0.0001; 0 ± 0 ng/mL). Donor milk CS content was also significantly higher than formula (#*p* < 0.0111).

### 3.3 Purity of chondroitin sulfate

**FIGURE 3:**
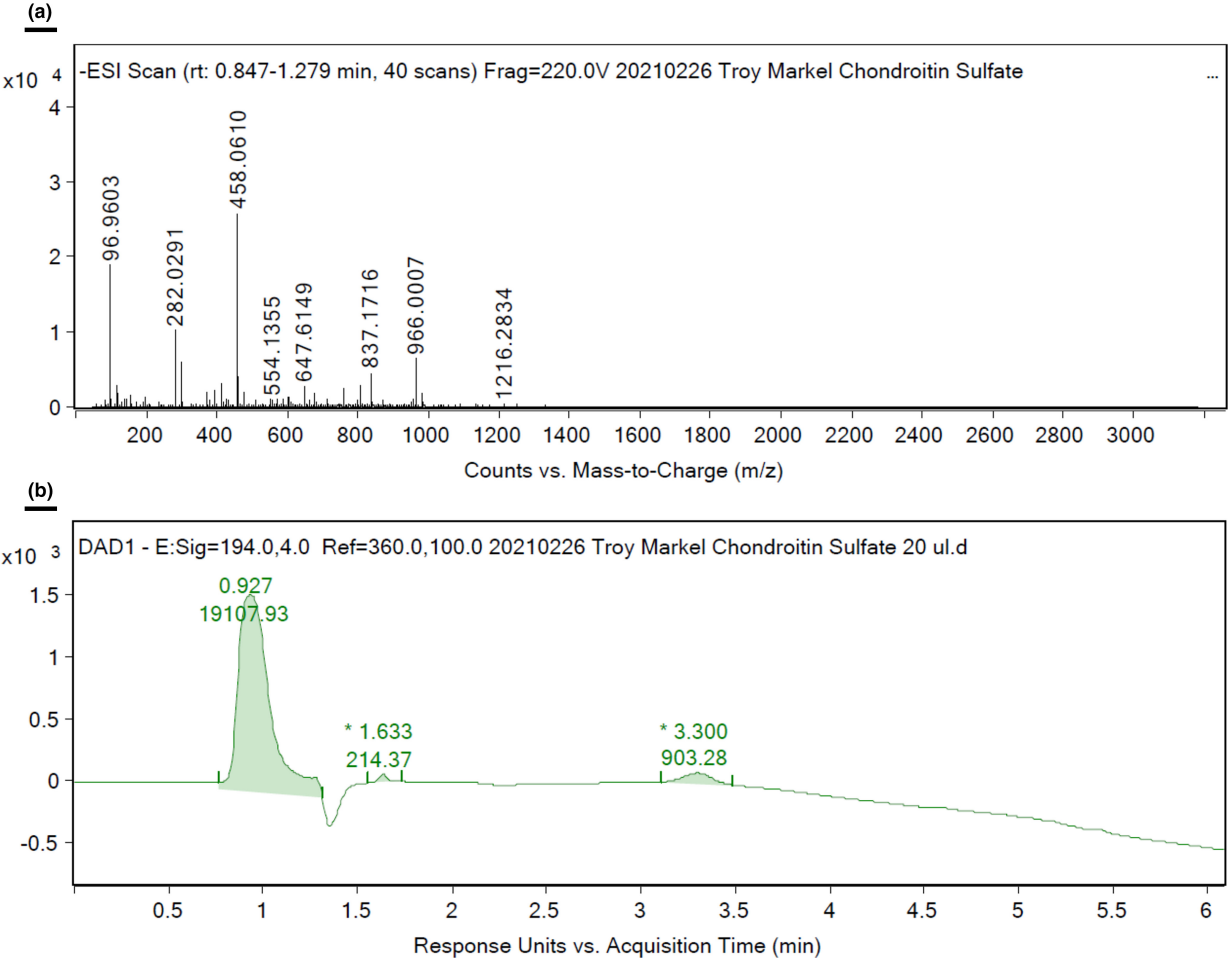
Chondroitin sulfate (CS) purity. An analysis of CS was performed on Agilent 1290 LC‐6545 QTOF. (a) Chondroitin sulfate unit under ESI‐negative ionization mode. The identity of CS was confirmed by QTOF under negative ionization mode, and mass signal m/z 458.0610 was observed as the Chondroitin sulfate unit. (b) Chondroitin sulfate purity under LC‐UV 194 nm. The purity of CS was established by a UV detector under 194 nm. CS eluted at a retention time of 0.927 min was 94.5% pure.

The purity of the CS used in animal experiments was validated and the results are shown in Figure 3. The identity of a single sulfated CS was confirmed by QTOF under negative ionization mode with mass signals m/z 458.0610 observed as the Chondroitin Sulfate unit (Figure 3a). The largest peak purity of CS was at 94.5% under a UV detector under 194 nm (Figure 3b).

### 3.4 Chondroitin sulfate supplementation improved outcomes in experimental NEC

**FIGURE 4:**
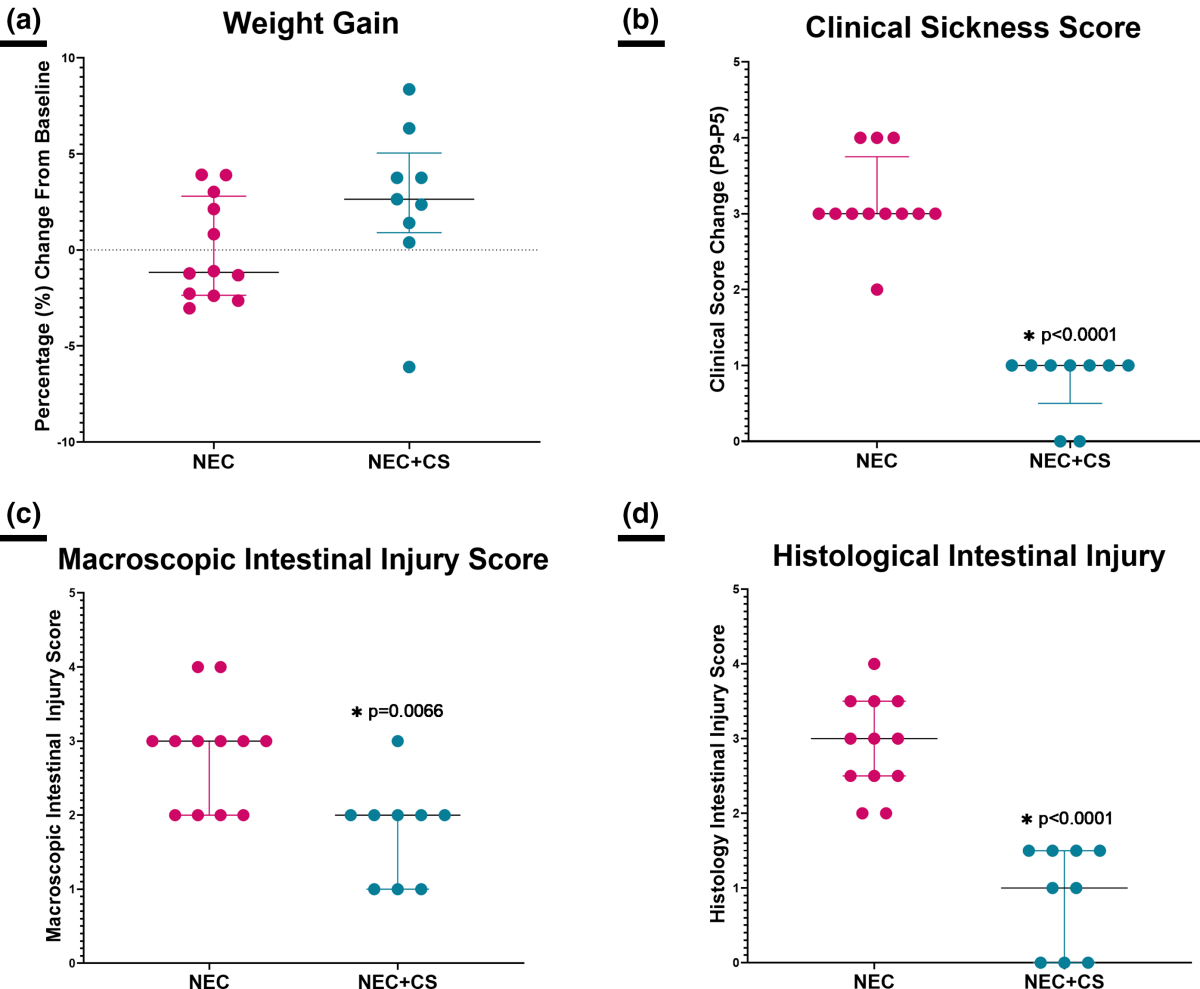
Necrotizing enterocolitis (NEC, *n* = 12) versus necrotizing enterocolitis + CS in formula (NEC + CS, *n* = 9; a) Weight Change: Mice with NEC+ CS supplementation (NEC + CS) showed no significant difference in weight gain compared to mice with NEC alone. (b) Clinical Sickness Score Change: Mice with NEC + CS showed a significant improvement in clinical sickness scores when compared to NEC alone (*p* < 0.0001; c) Macroscopic Gut Injury Score: Mice with NEC + CS showed a significant improvement of macroscopic gut injury scores when compared to NEC alone (*p* = 0.0066; d) Microscopic Intestinal Histology Score: Mice with NEC + CS showed a significant improvement of microscopic intestinal histology scores when compared to NEC alone (*p* < 0.0001). Mann–Whitney tests were performed.

When compared to NEC alone (*n* = 12), mice that underwent experimental NEC with CS‐supplemented formula (*n* = 9), had no significant difference in weight (NEC = −1.163[−2.358–2.802], NEC + CS = 2.642 [0.9003–5.049], *p = 0.1071, Figure 4a).

When compared to NEC alone, mice that underwent experimental NEC with CS‐supplemented formula showed improved clinical severity scores (NEC = 3 [3–3.75], NEC + CS = 1[0.5–1], *p < 0.0001, Figure 4b), lower macroscopic intestinal injury scores (NEC = 3 (Bazacliu & Neu, 2019; Neu & Walker, 2011), NEC + CS = 2 (Bazacliu & Neu, 2019; Jacob et al., 2015), *p = 0.0066, Figure 4c), and lower histological intestinal injury scores (NEC = 3 [2.5–3.5], NEC + CS = 1[0–1.5], *p < 0.0001, Figure 4d).

**FIGURE 5:**
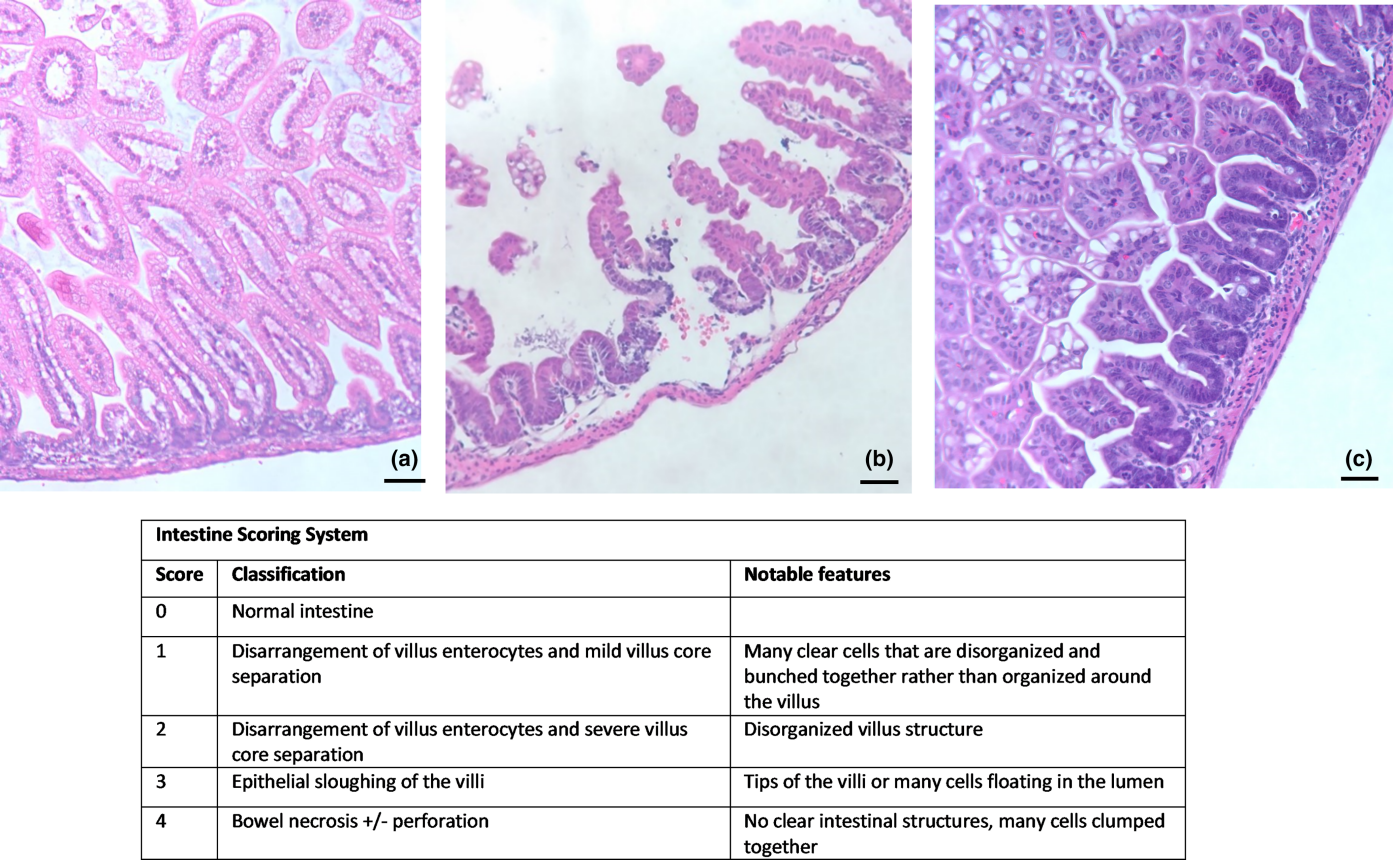
Representative intestinal histology. (a)Breastfed control intestine. This representative slice of an intestine from a breastfed control mouse pup showed a grade 0 score. (b) NEC intestine. This representative slice of an intestine from a mouse pup after experimental NEC showed grade 3 intestinal injury. (c) NEC + CS intestine. This representative slice of an intestine from a mouse pup after experimental NEC + CS showed grade 1 intestinal injury.

Representative histological sections are shown in Figure 5. The intestine of breastfed controls (Figure 5a) illustrated an intact basement membrane and intestinal crypts giving the specimen a histological score of “0.” The histological intestinal specimens of the NEC experimental model (Figure 5b) showed severe disruption of the basement membrane and crypts with floating villi in the lumen resulting in a histological score of “3.” Histological sections of mice undergoing NEC with supplementation by CS (Figure 5c) showed improvement of the effects seen in NEC with retainment of basement membrane and villus structures, resulting in a histological score of “1.”

### 3.5 Chondroitin sulfate did not significantly improve mortality

**FIGURE 6:**
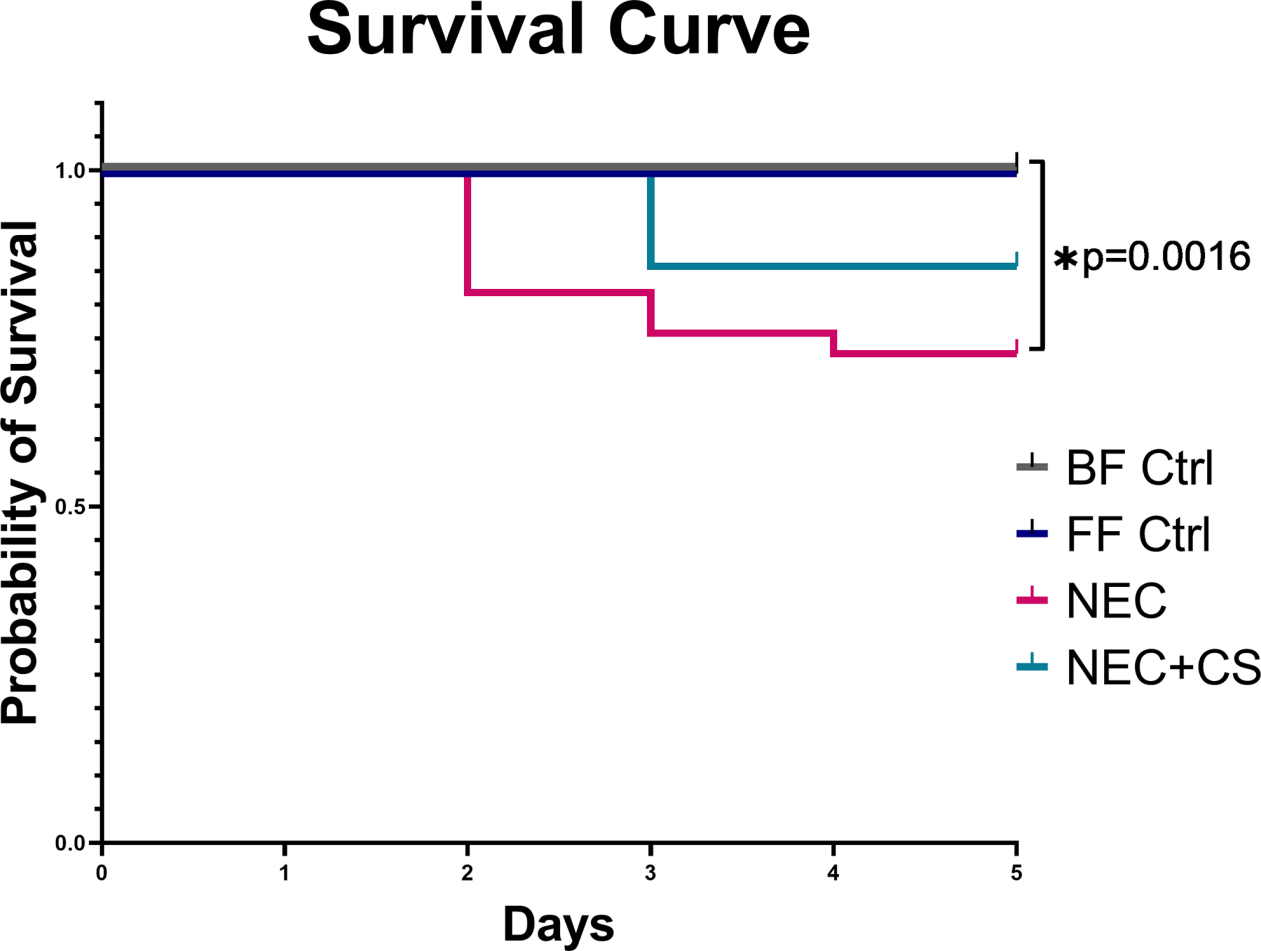
Mortality curves. Mortality curves for breastfed controls, formula‐fed controls, NEC experimental group, and NEC experimental group undergoing CS supplementation are shown. There was a significant difference between mortality curves using log‐rank Gehan–Breslow–Wilcoxon test between BF Ctrl and NEC curves (*p* = 0.0016) with Bonferroni's corrected value *p* < 0.0083 considered significant. No other significant differences between curves.

Mortality curves for breastfed controls, formula‐fed controls, NEC experimental group, and NEC experimental group undergoing CS supplementation are shown in Figure 6. There was a significant difference between curves using log‐rank (Mantel‐Cox) test (*p* = 0.0125). Multiple comparisons between groups with a Gehan–Breslow–Wilcoxon test and Bonferroni adjusted p< 0.0125 for significance revealed that there was a greater mortality in animals undergoing NEC alone when compared to breast‐fed‐controls (*p = 0.0016). There was no significant difference in mortality curves between breast‐fed and formula‐fed controls, between breast‐fed controls and animals undergoing NEC treated with CS, and between animals undergoing NEC and animals undergoing NEC treated with CS.

### 3.6 Chondroitin sulfate therapy was not affected by genetic ablation of eNOS

**FIGURE 7:**
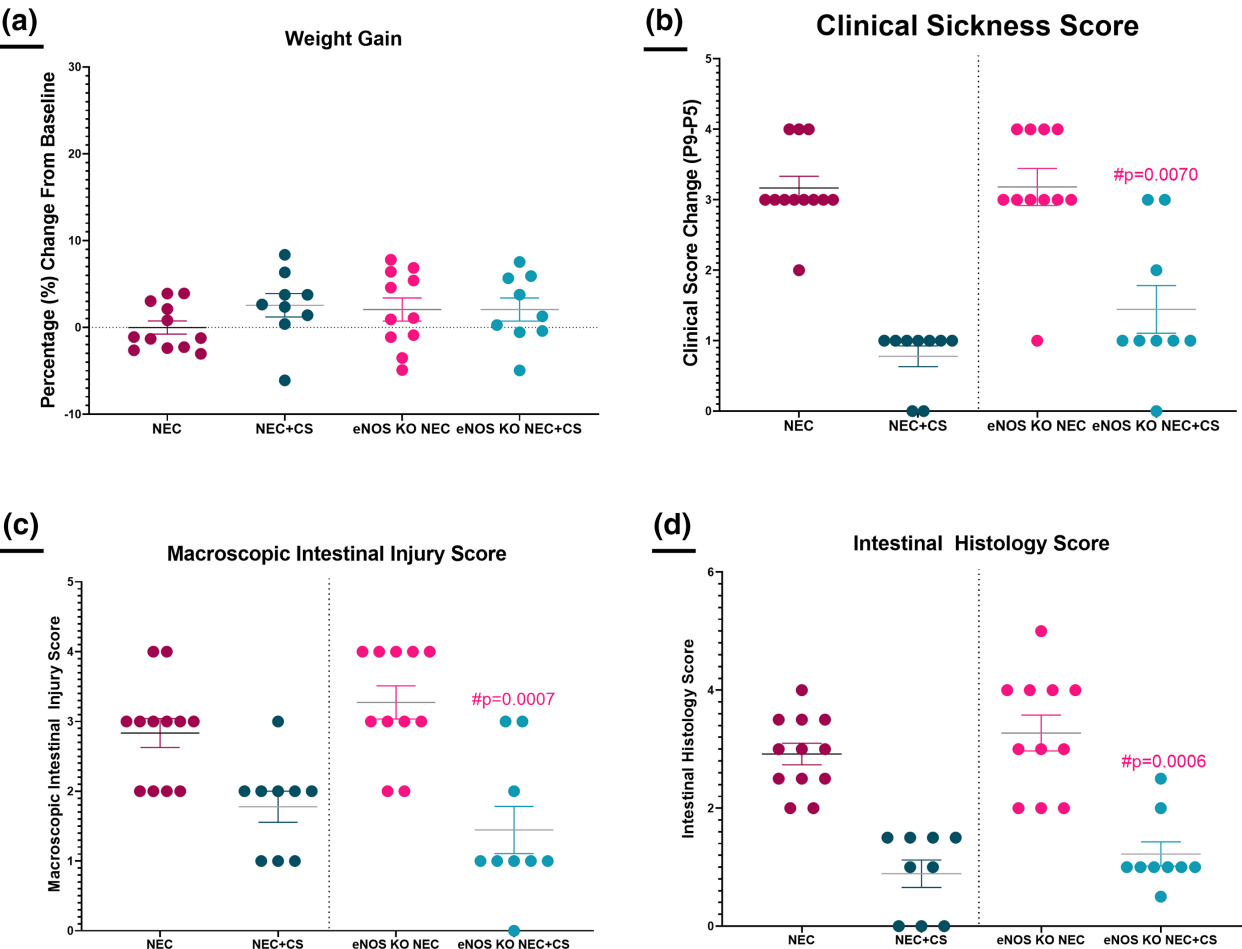
The effect of eNOS KO. (NEC, *n* = 12) versus (NEC + CS, *n* = 9) versus (eNOS NEC, *n* = 11) versus (eNOS NEC + CS, *n* = 9; a) Weight Change. No difference in weight between groups. *ANOVA* (b) Clinical Score Change. CS administration improves clinical scores between eNOS KO NEC + CS and eNOS KO NEC mice (#*p* = 0.0070). (c) Macroscopic Intestinal Injury Score. Significant improvement in eNOS KO + CS in macroscopic intestinal injury scores compared to the NOS KO group alone (#*p* = 0.0007). (d) Intestinal Histology Score. Significant improvement in eNOS KO + CS in histologic intestinal injury scores compared to the NOS KO group alone (#*p* = 0.0006). Kruskal–Wallis and ANOVA were performed as appropriate.

The previous cohort of WT mice undergoing NEC both with and without CS supplementation were compared to a new cohort of eNOS KO mice that underwent the NEC model both without CS (eNOS KO NEC, *n* = 11) and with CS (eNOS KO NEC + CS, *n* = 9) supplementation. There was no significant differences in weight gain between groups (eNOS KO NEC = 2.059 ± 1.326; eNOS KO NEC + CS = 2.060 ± 1.327, *p* = 0.3965 | Figure 7a).

In eNOS KO groups undergoing experimental NEC, there was a significant improvement with CS supplementation in clinical sickness scores (eNOS KO NEC = 3 (Knowles et al., 2021; Neu & Walker, 2011), eNOS KO NEC + CS = 1[1–2.5], #*p* = 0.0070 | Figure 7b), macroscopic intestinal injury scores (eNOS KO NEC = 3 (Knowles et al., 2021; Neu & Walker, 2011), eNOS KO NEC + CS = 1[1–2.5], #*p* = 0.0007 | Figure 7c), and intestinal histology scores (eNOS KO NEC = 3 (Bazacliu & Neu, 2019; Knowles et al., 2021; Neu & Walker, 2011), eNOS KO NEC + CS = 1[1–1.5], #*p* = 0.0006 | Figure 7d).

There were no differences between eNOS KO and WT groups undergoing NEC alone in clinical sickness scores (*p* > 0.9999, Figure 7b), macroscopic intestinal injury scores (*p* = 0.9678, Figure 7c), and histological intestinal injury scores (*p* > 0.9999, Figure 7d).When comparing outcomes between eNOS KO and WT groups receiving CS supplementation (NEC + CS vs eNOS KO NEC + CS), there were no significant differences in clinical sickness scores (*p* = 0.9464, Figure 7b), macroscopic intestinal injury scores (*p* > 0.9999, Figure 7c), or histological intestinal injury scores (*p* > 0.9999, Figure 7d).

### 3.7 Microbial diversity of stool and CS treatment

**FIGURE 8:**
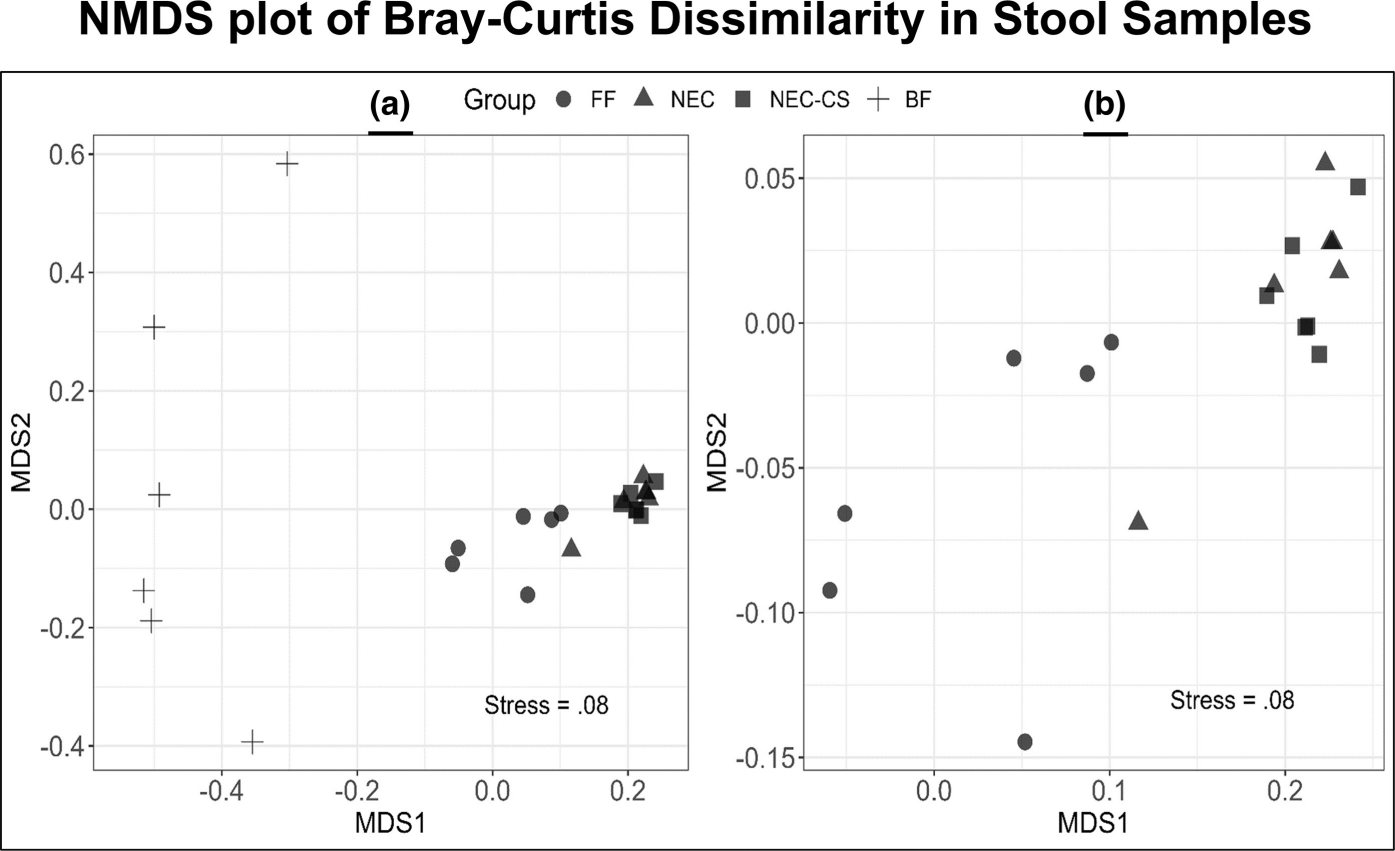
Bray–Curtis dissimilarity values: Nonmetric multidimensional scaling (NMDS) was then employed to look at the Bray–Curtis dissimilarity values (Figure 8) between groups in two dimensions with a stress value of 0.08 (stress values less than 0.1 are considered pretty good models for comparison; Song et al., 2017). In Figure 8a, breastfed controls (BF) were very different from each other and the other groups with abundant inter/intragroup variation BF Ctrl versus FF Ctrl (*p* = 0.030), versus NEC (*p* = 0.012), and versus NEC + CS (*p* = 0.018). Figure 8b is a zoomed‐in version of 7A that focuses on the remainder of the samples and showed two overall distinct “clusters” of samples: (1) formula‐fed samples and (2) NEC and NEC‐CS samples. Formula‐fed samples were significantly different from NEC samples (*p* = 0.048) and NEC + CS samples (*p* = 0.018). NEC and NEC + CS groups had the most overlap with each other (*p* = 1). PERMANOVA analysis performed.

Nonmetric multidimensional scaling (NMDS) was employed to look at the Bray–Curtis dissimilarity values (Figure 8) between the microbial diversity present in the stool of breastfed controls (BF Ctrl *n* = 6), formula‐fed controls (FF Ctrl *n* = 6), mice undergoing NEC (NEC, *n* = 6), and mice undergoing NEC with CS supplementation (NEC + CS, *n* = 6). This was compared in two dimensions with a stress value of 0.08 (stress values less than 0.1 are considered good models for comparison; Song et al., 2017). In Figure 8a, breastfed controls (BF) were very different from each other and the other groups with abundant inter and intragroup variation (BF Ctrl v FF Ctrl [*p* = 0.030], v NEC [*p* = 0.012], v NEC + CS [*p* = 0.018]). Figure 8b is a zoomed‐in version of 8a that focuses on the remainder of the samples and showed two overall distinct “clusters” of samples: (1) formula‐fed samples and (2) NEC and NEC‐CS samples. Formula‐fed samples were significantly different from NEC samples (*p* = 0.048) and NEC + CS samples (*p* = 0.018). NEC and NEC + CS groups had the most overlap with each other (*p* = 1) or the least dissimilarity from each other.

### 3.8 There was no significant improvement in NEC dysbiosis with supplementation of CS

**FIGURE 9:**
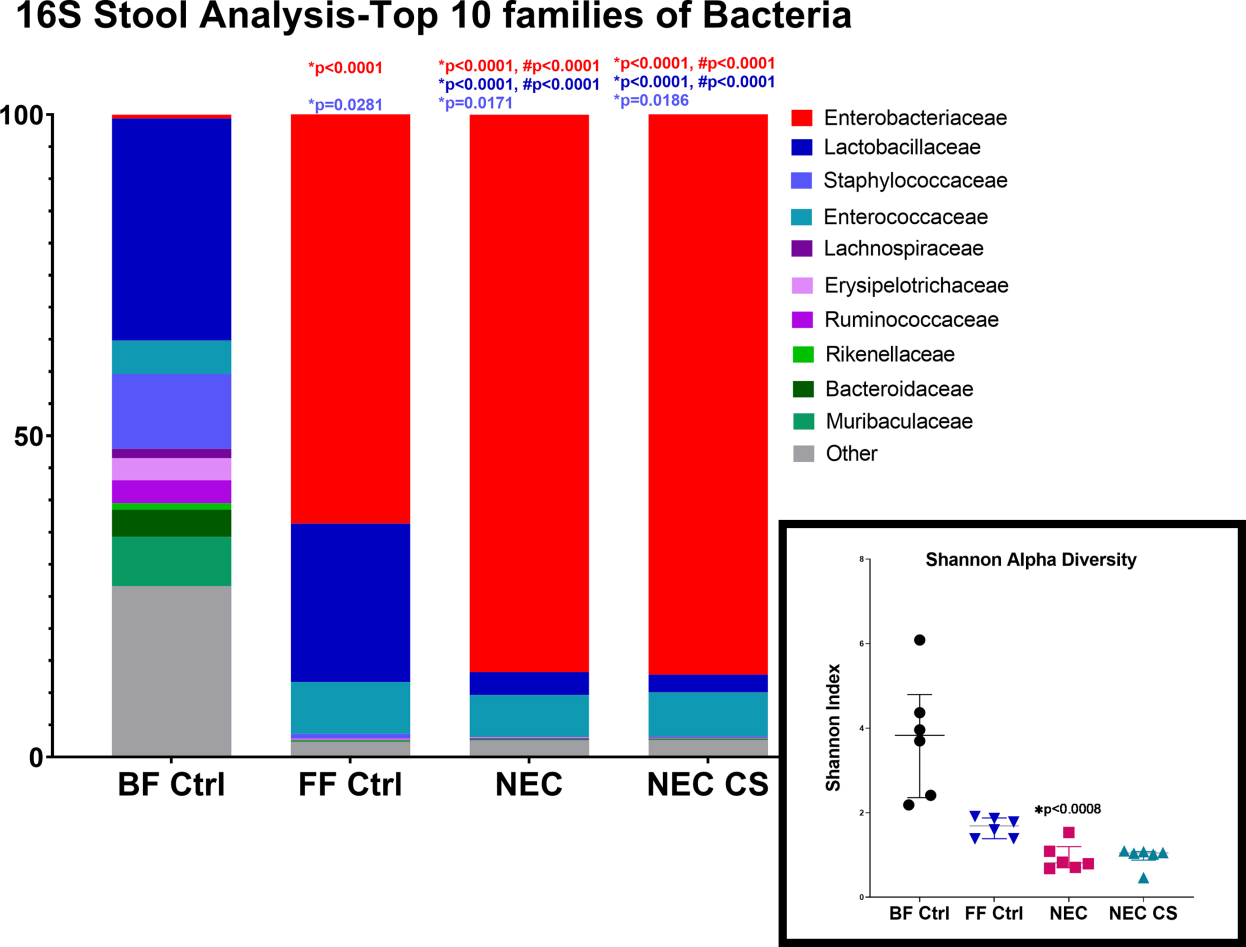
16S Ribosomal stool analysis: Top 10 families of Bacteria (*n* = 6 samples per group): Enterobacteriaceae family When compared to BF Ctrl, there was a significant increase in FF Ctrl, NEC, and NEC + CS (*p < 0.0001). When compared to FF Ctrl, there was a significant increase in this population in NEC and NEC + CS groups (#*p* < 0.0001). No difference between NEC and NEC + CS groups. Lactobacillaceae family: When compared to BF Ctrl, there was a significant decrease in NEC and NEC + CS groups (*p < 0.0001). When compared to FF Ctrl, there was a significant decrease in NEC and NEC + CS groups (#*p* < 0.0001). No difference between NEC and NEC + CS groups. Staphylococcaceae family: When compared to BF Ctrl, there was a significant decrease in FF Ctrl (*p = 0.0281), NEC (*p = 0.0171), and NEC + CS (*p = 0.0186). No difference between NEC and NEC + CS groups. Shannon Index of Alpha Diversity Mice with NEC showed a decreased level of alpha diversity when compared to breastfed controls (*p = 0.0008) but not formula‐fed controls. No significant difference between BF and FF Ctrl. No significant difference was seen between NEC and NEC + CS groups. Two‐way Kruskal–Wallis analyses were performed.

To further breakdown the microbial diversity, a 16‐s rRNA stool analysis was performed that showed the top 10 families of bacteria present in the stool between breastfed controls (BF Ctrl *n* = 6), formula‐fed controls (FF Ctrl *n* = 6), mice undergoing NEC (NEC, *n* = 6), and mice undergoing NEC with CS supplementation (NEC + CS, *n* = 6). These are shown in Figure 9 as a percentage out of 100% and a two‐way ANOVA was performed for analysis.

When compared to breastfed controls, there was a significant increase in the *Enterobacteriaceae* family population in formula‐fed controls (FF Ctrl, *p < 0.0001, 95% CI [−73.47, −52.62]), NEC alone (NEC, *p < 0.0001, 95% CI [−96.60, −75.75]), and NEC with CS supplementation (NEC + CS, *p < 0.0001, 95% CI [−96.97, −76.12]). Similarly, when compared to formula‐fed controls, there was a significant increase in NEC (NEC, #*p* < 0.0001, 95% CI [−33.55, −12.70]) and NEC with CS supplementation (NEC + CS, #*p* < 0.0001, 95% CI [−33.93, −13.08]). However, there was no difference in the population presence of *Enterobacteriaceae* between the mice undergoing NEC alone and those undergoing NEC with CS supplementation.

When compared to breastfed controls, there was a significant decrease in the *Lactobacillaceae* family populations in the NEC group (NEC, *p < 0.0001, 95% CI [20.57, 41.42]) and NEC with CS supplementation group (NEC + CS, *p < 0.0001, 95% CI [21.35, 42.21]. Similarly, when compared to formula‐fed controls, there was a significant decrease in the NEC group (NEC, #*p* < 0.0001, 95% CI [10.71, 31.56]) and the NEC with CS supplementation group (NEC + CS, #*p* < 0.0001, 95% CI [11.49, 32.34]). There was no significant difference in the *Lactobacillaceae* population in between formula‐fed and breastfed controls. Additionally, there was no difference in the population presence of *Lactobacillaceae* between the mice undergoing NEC alone and those undergoing NEC with CS supplementation.

When compared to breastfed controls, there was a significant decrease in the *Staphylococcaceae* family, in formula‐fed controls (FF Ctrl, *p = 0.0281, 95% CI [0.5655, 21.42]), the NEC group (NEC, *p = 0.0171, 95% CI [1.243, 22.10]), and in the NEC with CS administration group (*p = 0.0186, 95% CI [1.132, 21.98]. However, there was no difference in the population presence of *Staphylococcaceae* between the mice undergoing NEC alone and those undergoing NEC with CS supplementation.

Although there was no significant difference in populations of other bacteria between groups, it is important to note that the overall diversity when looking at the composition breakdown was decreased in the NEC model when compared to the breastfed controls. This was quantified by the Shannon Alpha Diversity, which was significantly decreased in the NEC experimental group when compared to breastfed controls (*p = 0.0008) with no significant difference seen when compared to formula‐fed controls (*p* = 0.1219). There was no significant difference in Shannon alpha diversity between breastfed and formula fed controls (*p* = 0.4833). There was no significant recovery in Shannon alpha diversity with CS supplementation when compared to NEC alone (*p* > 0.9999; BF Ctrl = 3.830 [2.356–4.798], FF Ctrl = 1.689 [1.386–1.875], NEC = 0.8127[0.6982–1.1199], and NEC + CS = 1.047 [0.8791–1.081] | Figure 9).

### 3.9 CS improves cytokine profile in intestinal segments

**FIGURE 10:**
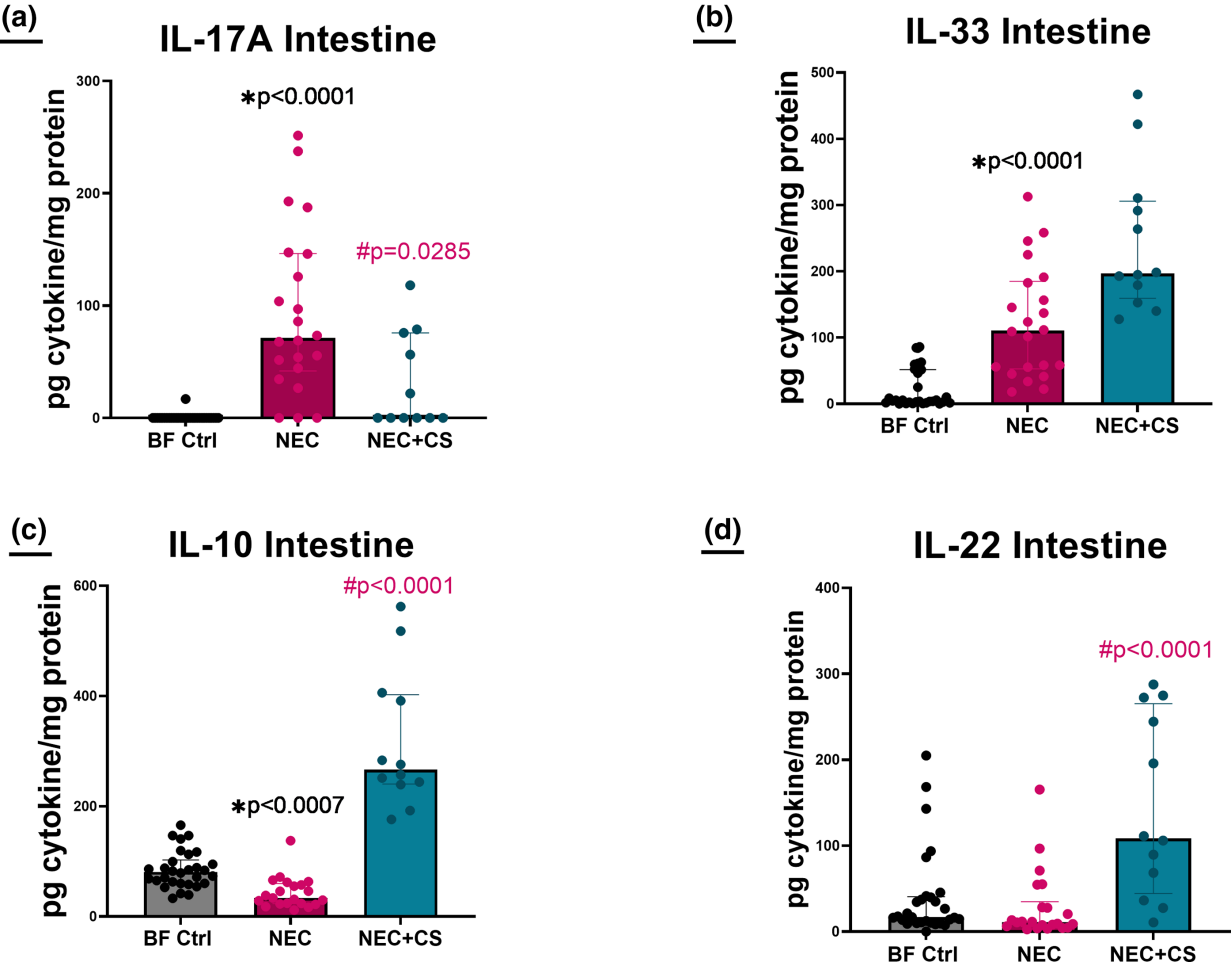
CS improves cytokine profile in intestinal segments (n?). Multiplex beaded assay was used to analyze homogenized intestinal segments between BF Ctrl (*n* = 30), NEC (*n* = 22), and NEC + CS (*n* = 11). Significant differences were seen in IL‐17A, IL‐22, IL‐10, and IL‐33 levels. IL‐17A levels were significantly elevated in NEC compared to breastfed controls (*p < 0.0001) and significantly decreased in NEC + CS compared to NEC alone (#*p* = 0.0285; [BF Ctrl = 0[0–0], NEC = 71.20[41.83–146.4], NEC + CS = 0[0–75.79]; Figure 10a). IL‐33 levels were significantly elevated in NEC compared to breastfed controls (*p < 0.0001) with no significant change between NEC + CS compared to NEC alone (*p* = 0.0616; [BF Ctrl = 5.05[1.71–51.7], NEC = 110.4[52.8–184.8], and NEC + CS = 196.6[159.3–305.7] Figure 10b). IL‐10 levels were significantly decreased in NEC compared to breastfed controls (*p = 0.0007) and significantly increased in NEC + CS compared to NEC alone (#*p* < 0.0001; [BF Ctrl = 80.64[60.10–102.8], NEC = 33.61[23.38–59.77], and NEC + CS = 266.5[240.5–402.5] Figure 10c). There was no difference in IL‐22 levels in NEC compared to breastfed controls (*p* = 0.2430) but was significantly increased in NEC + CS compared to NEC alone (#*p* < 0.0001; [BF Ctrl = 16.93[10.56–40.62], NEC = 11.12[5.780–34.88], and NEC + CS = 108.6[44.8–265.3] Figure 10d). Kruskal–Wallis analyses were performed with Dunn's multiple comparisons test.

Multiplex beaded assay was used to analyze homogenized intestinal segments between BF Ctrl (*n* = 30), NEC (*n* = 22), and NEC + CS (*n* = 11). Significant differences were seen in IL‐17A, IL‐22, IL‐10, and IL‐33 levels. IL‐17A levels were significantly elevated in NEC compared to breastfed controls (*p* < 0.0001) and significantly decreased in NEC + CS compared to NEC alone (#*p* = 0.0285; [BF Ctrl = 0[0–0], NEC = 71.20[41.83–146.4], and NEC + CS = 0[0–75.79]; Figure 10a). IL‐33 levels were significantly elevated in NEC compared to breastfed controls (*p < 0.0001) with no significant change between NEC + CS and NEC alone (*p* = 0.0616; [BF Ctrl = 5.05[1.71–51.7], NEC = 110.4[52.8–184.8], NEC + CS = 196.6[159.3–305.7] Figure 10b). IL‐10 levels were significantly decreased in NEC compared to breastfed controls (*p = 0.0007) and significantly increased in NEC + CS compared to NEC alone (#*p* < 0.0001; [BF Ctrl = 80.64[60.10–102.8], NEC = 33.61[23.38–59.77], and NEC + CS = 266.5[240.5–402.5] Figure 10c). There was no difference in IL‐22 levels in NEC compared to breastfed controls (*p* = 0.2430) but was significantly increased in NEC + CS compared to NEC alone (*p* < 0.0001; [BF Ctrl = 16.93[10.56–40.62], NEC = 11.12[5.780–34.88], and NEC + CS = 108.6[44.8–265.3] Figure 10d).

## 4 DISCUSSION

Necrotizing enterocolitis (NEC) remains a devastating disease of the newborn. Currently, there are no targeted medical therapies available to treat NEC, however, the importance of breast milk is well‐studied and documented (Knowles et al., 2021). Due to the disparities that limit access to breast milk (Knowles et al., 2021), it is important to identify the most important biological components of HM so that formula supplementation can occur for those who do not have access to HM.

Due to the emerging literature surrounding the benefits of chondroitin sulfate (CS) in several disease processes including colitis and IBD (Linares et al., 2015), as well as its higher concentrations in HM, we chose to study the potential benefit of CS therapy in NEC. Although the exact mechanisms and functions of glycosaminoglycans such as CS are incompletely understood, studies attributing their proposed protective effects to the pathophysiology of NEC are accumulating (Bering, 2018; Burge et al., 2019; Knowles et al., 2021). Our study supports the emerging data that CS could be a key component of breast milk in the protection against NEC. We observed that the supplementation of CS improved several outcomes in experimental NEC, including clinical sickness scores, macroscopic intestinal injury scores, histologic intestinal injury scores, and the intestinal tissue cytokine profile.

Knowing the beneficial effects of CS, we wanted to examine the differences in human breast milk, banked human donor milk, and formula. Our studies showed that the content of CS dropped significantly in the donor milk of mothers of term infants when compared to breast milk of mothers of term infants suggesting that the processing and storage process could affect CS content. Additionally, the CS content in the tested formulas was nonexistent. These data, in conjunction with the animal data from our murine experimental NEC model, support the need for supplementation of donor breast milk and formulas with CS as a necessary adjunct in the prevention and treatment of NEC.

Given that the sulfated residues of CS are believed to participate and regulate various biological functions (Anower & Kimata, 2015), as well as previous data from our lab suggesting that multiple other sulfur‐derived compounds exerted their beneficial properties through the eNOS pathway (Drucker, Jensen, Ferkowicz, & Markel, 2018; Hosfield et al., 2022; Jensen et al., 2017), we hypothesized that the effects of CS supplementation would also be mediated through this same pathway. The CS used for our experiments was bovine‐cartilage‐derived and mono‐sulfated, which was as expected from the literature (Martel‐Pelletier et al., 2015; Volpi, 2007). However, the end effects on tissue protection with CS supplementation did not appear to change when eNOS was ablated. This suggested that sulfur‐signaling via the eNOS pathway was not the primary means through which CS exerted its therapeutic effects in our experimental NEC model.

CS has been shown to decrease the invasion of pathogenic bacteria across the gut wall, promote the growth of commensal bacteria, and increase microbial diversity (Knowles et al., 2021). The microbiota of healthy term infants is characterized by increased diversity and an abundance of *Lactobacillus, Bifidobacteria*, and *Bacteroidetes*, whereas preterm infants show decreased diversity and dysbiosis toward more pathogenic bacteria such as of the *Enterobacteriaceae* family (Bering, 2018; Rinninella et al., 2019). Specifically, CS prevents bacterial invasion and translocation by interfering with the ability of these microbes to adhere to intestinal epithelial cells. In vitro studies by Burge et al. showed up to 75% reduction of bacterial invasion with 750 μg/mL concentration with no decrease in intestinal cell viability (Bering, 2018; Burge et al., 2019).

Our studies, however, showed that CS supplementation in formula did not significantly improve the dysbiosis and lack of diversity that occurs in NEC. Our data supported the literature and showed that NEC was associated with an increase in pathogenic species such as *Escherichia spp* as well as a decrease in commensal species such as *Lactobacillus spp* and *Staphylococcus spp* with an accompanying decrease in microbial diversity (indicated by Shannon alpha diversity). Interestingly, formula feeding also decreased microbial diversity and resulted in more dysbiosis when compared to breastfed controls, although not to the degree of NEC. When formula‐fed controls and NEC were compared, mice with NEC had significantly worse dysbiosis and decreased diversity. Our studies also showed that CS supplementation in formula did not significantly improve the dysbiosis and lack of diversity that occurs in NEC, suggesting that another mechanism is responsible for the beneficial outcomes seen with CS therapy in our model of experimental NEC.

Finally, when we looked at the cytokine profile of the intestinal segments, we saw an improvement in several inflammatory cytokines in the intestine. IL‐33 levels were significantly elevated in NEC compared to breastfed controls with no significant change with CS therapy. In the literature, IL‐33, is considered a biomarker of interest in NEC (Cakir et al., 2020). IL‐33 is an alarmin and an initial acute phase reactant. Although the exact role of IL‐33 in NEC is not understood, in colitis and IBD, IL‐33 has both a pathogenic role in immune activation as well as a protective role in modulating regulatory T cells. Our data show that mice with NEC have increased expression of IL‐33 which suggests that it serves as an alarmin and has some pathogenic role. However, treatment with CS did not decrease levels of IL‐33, and in fact, the trend suggests that CS may actually increase IL‐33 (Chen et al., 2020). Further research is needed on this cytokine to understand the duality of its role in this disease. NEC had increased IL‐17A levels compared to breastfed controls, and the group treated with CS undergoing NEC had a significant decrease in IL‐17A. IL‐17A has been implicated in many models of intestinal disease and is considered a biomarker of interest in NEC (Tremblay et al., 2021). Additionally, dysregulation of IL‐17A is hypothesized to be a driver for the inflammation in NEC (Lawrence et al., 2018), and thus IL‐17A has been considered a target of interest in this disease process (Wynn et al., 2016; Fauny et al., 2020). IL‐10 is an anti‐inflammatory cytokine (Hu et al., 2017) and levels were significantly decreased in NEC compared to breastfed controls as expected. Interestingly, treatment with oral CS significantly increased the levels of IL‐10. IL‐22 is implicated in mucosal healing (Mihi et al., 2021) and has been shown to promote epithelial cell regeneration in NEC (Mihi et al., 2021). There was also a significant increase in IL‐22 with CS therapy in experimental NEC. Overall, CS treatment seems to significantly improve the cytokine profile in the intestine by downregulating the inflammatory cytokine IL‐17A and upregulating protective anti‐inflammatory cytokines including IL‐10 and IL‐22.

Although much remains to be understood about the mechanisms of therapeutic effect behind CS, our findings clarify that the primary mechanisms through which CS exerts its effects in NEC are likely not through the eNOS pathway or through modifying the microbiome. Our findings instead support that CS may modulate the inflammatory cytokine signaling cascade. CS has been shown to participate in immunomodulation in other disease processes. In chondrocytes and osteoarthritis, CS has been shown to diminish the release of pro‐inflammatory cytokines such as TNF‐α or IL‐1β via NF‐ĸB translocation (Ade‐Ajayi et al., 1996; Drucker, Jensen, Ferkowicz, & Markel, 2018). In inflammatory bowel disease, NF‐ĸB is markedly induced in IBD patients as well as an accompanied increase in pro‐inflammatory cytokines, and CS is theorized to improve clinical symptoms by reducing activation of NF‐ĸB in intestinal mucosa (Volpi, 2007). Further studies using our murine model of NEC will need to be undertaken to determine the effect of CS therapy on immune cell populations in the intestine as well as systemic changes in immune cell populations and cytokine profile in other tissues. In addition, studies will need to be undertaken to determine the molecular pathways (such as NF‐ĸB pathway activation) through which CS may act have an immune‐modulatory effect.

## 5 LIMITATIONS

This study was limited by nature in that it was an animal model. As stated previously, NEC is a complex multifactorial disease and there are several variations of animal models that try to recapitulate the pathophysiology (Bazacliu & Neu, 2019). These models have deepened our understanding of the disease, but likely do not fully represent the underlying pathophysiology of the disease process. Additionally, although we determined that the CS used was primarily mono‐sulfated, our current LCMS data cannot tell the distribution of the lengths and position of the sulfated groups. We plan to repeat experiments with a pharmaceutical‐grade‐derived CS to ensure the reproducibility and biochemical nature of the chondroitin sulfate that we receive. In addition, in the study of the microbiome of term mice, it is important to understand that there may be key differences in the components of a normal microbiome when compared to preterm infants. Furthermore, although a stool analysis for microbial composition was done, the stool collected was in the rectal vault. It is possible that there could be more changes in the local microbiota along the proximal intestines and cecum that may not have been captured in our stool sampling and would be an interesting focus of a future study. Although no significant difference was found in alteration of microbial dysbiosis and diversity with the administration of CS in the NEC experimental model using our concentration, we do not know if there could be an effect with a higher dose or longer exposure to CS. Lastly, the 16S rRNA analysis performed was limited to the top 10 bacterial species, with most granularity existing down to the family level. This lends itself to the need for more advanced diagnostic tools such as whole‐genome sequencing of stool and intestinal segments to obtain a better understanding of the subtle effects and changes of the microbiome. Future studies are needed to determine the optimum dosing of chondroitin sulfate in humans needed to maximize clinical effects.

## 6 CONCLUSION

Chondroitin sulfate is common oral supplement that can be used for the prevention and treatment of necrotizing enterocolitis. Our findings indicate that it attenuated the severity of NEC by improving clinical and histological outcomes. Although the mechanisms of actions by which CS exerted these improvements remain to be elucidated, our data suggested that improvements do not occur through the eNOS pathway or by altering the diversity of the microbiome. CS may instead mediate immunomodulatory mechanisms that alter the cytokine profile. Further research is needed to identify additional mechanisms through which CS exerts its protective effects as well as to determine whether CS could be a safe and effective targeted medical therapy for NEC.

## AUTHOR CONTRIBUTIONS

Authors listed in manuscript are eligibe for authorship and take responsibility for particular sections of manuscript. The author contributions are individually clarified in the following statements. All authors critically reviewed the article and approved its final form. Study conception and design: Krishna Manohar, Brian D. Hosfield, and Troy A. Markel. Data acquisition: Krishna Manohar, Brian D. Hosfield, Lifan Zeng, and John P. Brokaw. Analysis and data interpretation: Krishna Manohar, Brian D. Hosfield, Cameron Colgate, Fikir M. Mesfin, W. Christopher Shelley, Jianyun Liu, and Troy A. Markel. Drafting of the article: Krishna Manohar. Critical revision: Troy A. Markel, John P. Brokaw, Brian D. Hosfield, Fikir M. Mesfin, Lifan Zeng, Jianyun Liu, W. Christopher Shelley, Cameron Colgate, and Krishna Manohar.

## FUNDING INFORMATION

Funding for this project was received from 1) National Institutes of Health (R01DK133418, R01HD105301), 2) American College of Surgeons Clowe's Memorial Research Fund, 3) Gerber Foundation, Chan Zuckerberg Initiative 2022‐316749, 4) Riley Children's Foundation, and 5) IU Department of Surgery.

## ETHICS STATEMENT

This study was conducted under the approval of the Indiana University Institutional Review Board (IRB protocol # 20114) and the Indiana University Institutional Animal Care and Use Committee (Protocol #19122).
